# An image fusion system for corrective osteotomy of distal radius malunion

**DOI:** 10.1186/s12938-021-00901-8

**Published:** 2021-06-30

**Authors:** Yuichi Yoshii, Takeshi Ogawa, Yuki Hara, Yasukazu Totoki, Tomoo Ishii

**Affiliations:** 1 Department of Orthopaedic Surgery, Tokyo Medical University Ibaraki Medical Center, 3-20-1 Chuo, Ami, Inashiki, Ibaraki 300-0395 Japan; 2 Department of Orthopaedic Surgery, University of Tsukuba Hospital, Tsukuba, Ibaraki 305-8576 Japan

**Keywords:** Computed tomography, Corrective osteotomy, Distal radius malunion, Fluoroscopy, Image fusion, Preoperative plan

## Abstract

**Background:**

To provide surgical support for corrective osteotomy, we developed an image fusion system for three-dimensional (3D) preoperative planning and fluoroscopy. To assess the utility of this image fusion system, we evaluated the reproducibility of preoperative planning for corrective osteotomy of dorsally angulated distal radius malunion using the system and compared reproducibility without using the system.

**Methods:**

Ten wrists from 10 distal radius malunion patients who underwent corrective osteotomy were evaluated. 3D preoperative planning and the image fusion system were used for the image fusion group (n = 5). Only 3D preoperative planning was used for the control group (n = 5). 3D preoperative planning was performed for both groups in order to assess reduction, placement, and the choice of implants. In the image fusion group, the outline of the planned image was displayed on a monitor and overlapped with fluoroscopy images during surgery. Reproducibility was evaluated using preoperative plan and postoperative 3D images. Images were compared with the 3D coordinates of the radial styloid process (1), the volar and dorsal edges of the sigmoid notch (2) (3), and the barycentric coordinates of the three reference points. The reproducibility of the preoperative plan was evaluated by the distance of the coordinates between the plan and postoperative images for the reference points.

**Results:**

The distances between preoperative planning and postoperative reduction in the image fusion group were 2.1 ± 1.1 mm, 1.8 ± 0.7 mm, 1.9 ± 0.9 mm, and 1.4 ± 0.7 mm for reference points (1), (2), (3), and the barycenter, respectively. The distances between preoperative planning and postoperative reduction in the control group were 3.7 ± 1.0 mm, 2.8 ± 2.0 mm, 1.7 ± 0.8 mm, and 1.8 ± 1.2 mm for reference points (1), (2), (3), and the barycenter, respectively. The difference in reference point (1) was significantly smaller in the image fusion group than in the control group (P < 0.05).

**Conclusion:**

Corrective osteotomy using an image fusion system will become a new surgical support method for fracture malunion.

*Trial registration* Registered as NCT03764501 at ClinicalTrials.gov.

## Background

Distal radius malunion is a common complication of distal radius fractures. It occurs when a fracture of the distal radius heals with improper alignment, incorrect length, articular incongruity, or a combination of these factors. The incidence of distal radius malunion was previously reported to be approximately 5–24% of distal radius fractures [1–4]. Most cases of distal radius malunion are symptomatic due to changes in biomechanical conditions. It causes pain, weakness, or functional impairment at the wrist joint [1, 5–7]. Symptomatic distal radius malunion requires corrective osteotomy. The aim of corrective osteotomy is to reduce pain and improve wrist function. It needs to restore normal wrist joint congruency and realign the distal radius.

Preoperative planning for corrective osteotomy has traditionally been performed using two-dimensional (2D) radiographs of the anterior–posterior and lateral views [8–10]. However, conventional X-rays cannot sufficiently evaluate rotational deformities [9, 11, 12]. The outcomes of corrective osteotomy for rotational corrections using 2D imaging are mostly unsatisfactory [11]. In recent years, three-dimensional (3D) planning, navigation, and the possibility of implant customization have led to changes in the clinical practice of corrective osteotomy for distal radius malunion. 3D preoperative planning visualizes the reduction process and placement/choices of implants. It is particularly useful for visualizing the correction of rotational deformities [10, 13]. These innovations have been possible due to advances in the field of computer-assisted technologies. Although significant advances have been made in 3D visualization and planning, the use of 2D to 3D or 3D to 2D image conversion in clinical practice has not yet been perfected. To more smoothly perform 2D–3D conversion for osteosynthesis and osteotomy, we developed an image fusion system for 3D preoperative planning and fluoroscopy. This system draws an outline of a 3D preoperative planning image and projects it onto a fluoroscopic image [14]. To assess the utility of the image fusion system, we evaluated the reproducibility of preoperative planning in corrective osteotomy for dorsally angulated distal radius malunion using the system. The results obtained were compared to the reproducibility of a preoperative plan without using the system.

## Results

**Fig. 1:**
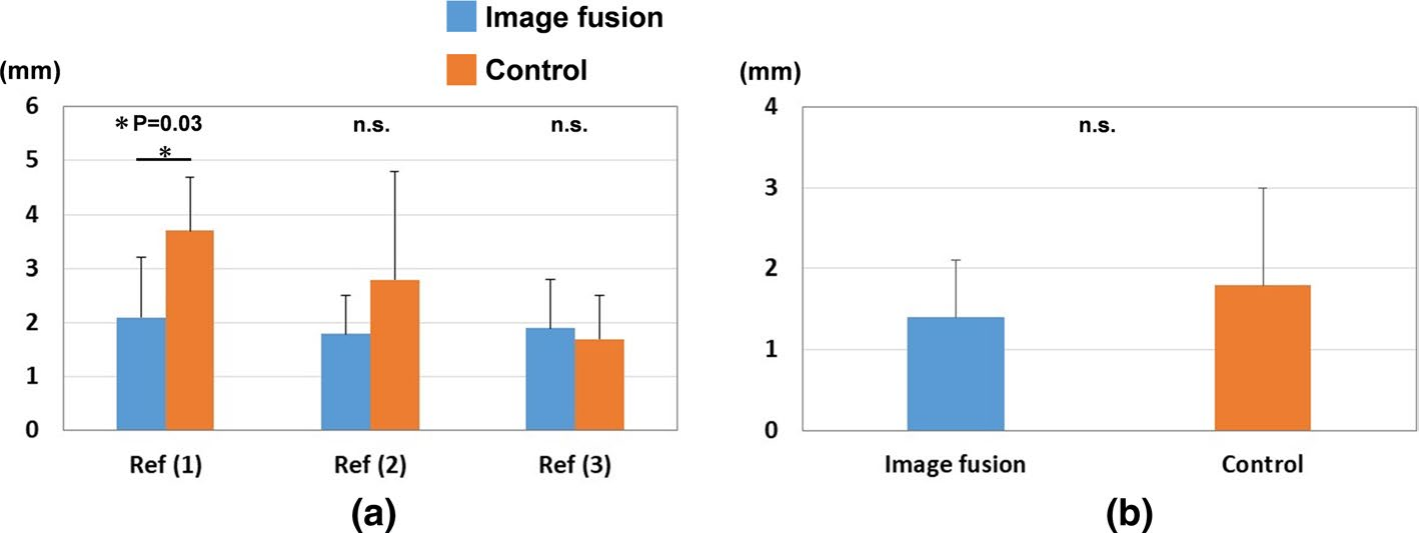
Results of correction accuracy for reference points. **a** Results for each reference point. Ref (1): the radial styloid process, Ref (2): the sigmoid notch volar edge, Ref (3): the sigmoid notch dorsal edge. (b) Results for the barycenter of the three reference points. Blue bars indicate distances between preoperative planning and postoperative reduction in the image fusion group. Orange bars indicate distances between preoperative planning and postoperative reduction in the control group. *: P = 0.03, n.s.: not significant

The results of correction accuracy for the reference points are shown in Fig. 1. The distances between preoperative planning and postoperative reduction in the image fusion group were 2.1 ± 1.1 mm, 1.8 ± 0.7 mm, 1.9 ± 0.9 mm, and 1.4 ± 0.7 mm for reference points (1), (2), (3), and the barycenter, respectively. The distances between preoperative planning and postoperative reduction in the control group were 3.7 ± 1.0 mm, 2.8 ± 2.0 mm, 1.7 ± 0.8 mm, and 1.8 ± 1.2 mm for reference points (1), (2), (3), and the barycenter, respectively. The difference in reference point (1) was significantly smaller in the image fusion group than in the control group (P < 0.05).

**Fig. 2:**
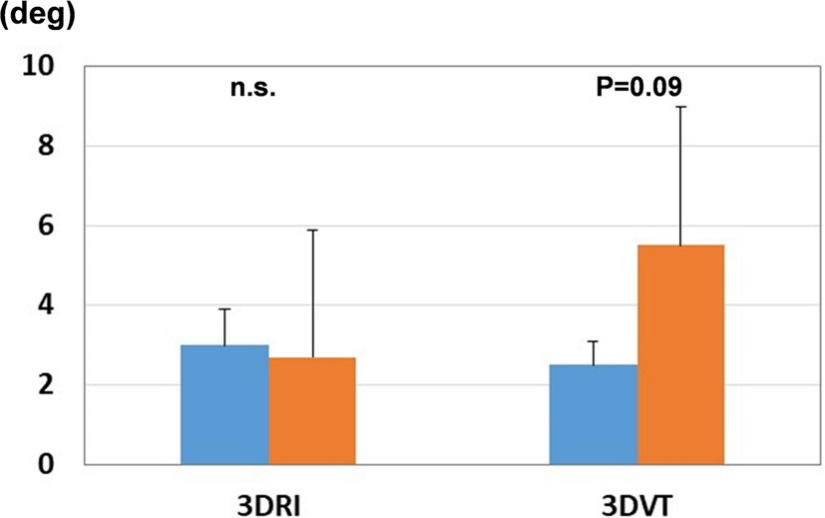
Results of correction accuracy for 3DRI and 3DVT. Blue bars indicate differences between preoperative planning and postoperative reduction in the image fusion group. Orange bars indicate distances between preoperative planning and postoperative reduction in the control group. n.s.: not significant

The results of correction accuracy for radial inclination and volar tilt on 3D images (3DRI, 3DVT) are shown in Fig. 2. Differences between the preoperative and postoperative plans in the image fusion group were 3.0 ± 0.9° and 2.5 ± 0.6° for 3DRI and 3DVT, respectively, while those in the control group were 2.7 ± 3.2° and 5.5 ± 3.5° for 3DRI and 3DVT, respectively. Small differences in 3DVT were observed in the image fusion group (P = 0.09). Mayo wrist scores were 52.5/58.8 and 78.8/68.8 (image fusion group / control group) in three and six months after surgery, respectively.

## Discussion

The present study evaluated the utility of our image fusion system in corrective osteotomy for distal radius malunion. A 3D simulation in combination with a fluoroscopic image is a novel surgical approach for corrective osteotomy. The image fusion system allows us to directly compare preoperative planning images with fluoroscopy images. With a visual guide during the surgical process, the surgeon had more confidence regarding implant placement and osteotomy. As a result, the reproducibility of the 3D preoperative plan for corrective osteotomy improved.

Since it is difficult to carry out a corrective osteotomy plan during actual surgery, several computer-aided techniques have been introduced [13]. With the spread of analytical software and 3D printers, the 3D visualization of osteotomy and preoperative evaluations are becoming more common [15]. Previous studies reported the use of optical tracking devices and synthetic templates to reproduce reduction shapes [16–19]. More recently, the possibility of computer assistance for corrective osteotomy by creating patient-specific surgical cutting guides and fixation plates was reported [20, 21]. The use of patient-specific guides is currently considered to be the most accurate method, but involves extra costs to perform surgery. Furthermore, positioning errors in the patient-specific guide affect correction accuracy [22]. The image fusion system allows the 3D preoperative plan to be directly compared with the fluoroscopic image without additional medical expenses. This is one of the benefits of using the image fusion system.

The reduction position of each reference point was reproducible with an error of 1.9–2.1 mm using the image fusion system. Previous studies showed a reduction accuracy of less than 2 mm when computer-aided technology was used in osteotomy for distal radius malunion [23–25]. Patient-specific instruments were used in these studies. Other studies demonstrated that in conventional osteotomy, only 40% of corrections achieved within 5 degrees of the planned correction for the angular deformity and within 2 mm of the planned ulnar variance [26]. The image fusion system does not require specific instruments. Nevertheless, the reduction accuracy of each reference point was similar to that obtained using patient-specific instruments. The difference between preoperative plan and postoperative reduction for the reference point (1) was slightly larger than other reference points because distal radius malunion is mostly deformed in the dorsal, radial and proximal directions. Therefore, the amount of movement for the radial styloid process becomes larger than other reference points. This difference tended to increase if the reduction could not be achieved accurately. Considering the improved reproducibility of reference point (1) and 3DVT, this may represent a strategy for improving reduction accuracy without using patient-specific instruments. Therefore, we propose this approach as a novel method for performing corrective osteotomy.

There were several limitations that need to be addressed. This was a case control study without randomization. The control group was enrolled from previous cases. Randomized controlled trials may be difficult to conduct due to the limited number of cases in a single facility. Large-scale investigations, such as case registration, may be needed to evaluate true practicality. Although image fusion in clinical medicine is already a well-known technology, there are two novelties in the present study: the use of an image fusion method in corrective osteotomy and the conversion of a 3D preoperative plan into a 2D contour extraction image, thereby allowing existing fluoroscopy to be used to perform image fusion. Since this is a preliminary study, the number of cases was limited. In this study, we attempted to establish a protocol for corrective osteotomy using the image fusion system. If this method is accepted, we plan to adapt it to more cases and verify its clinical significance. Another limitation is that the implementation of this method requires specialized software and proficiency in the procedure. There may be a learning curve and improved accuracy following the mastery of the procedure. This method also involves CT scans. In the present study, axial CT images with a slice thickness of 1–1.5 mm were used. Radiation exposure needs to be reduced by increasing the slice thickness or changing imaging conditions. Furthermore, no significant differences were observed in clinical outcomes. In a previous meta-analysis, 3D-planned corrective osteotomy significantly improved both radiographical and functional outcomes over the preoperative condition. However, it is still unclear whether these computer-assisted technologies improve clinical outcomes. This method may help residents and fellows who are unfamiliar with corrective osteotomy to visualize surgery and share information with other medical staff. In addition, the evaluation method presented herein may be applied to the biomechanical analysis of distal radius fractures. These educational and academic significances need to be investigated in future studies.

## Conclusions

An image fusion system was developed for corrective osteotomy. 3D reference points were reproducible with an error of approximately 2 mm in corrective osteotomy for distal radius malunion using the system. The reproducibility of the 3D preoperative plan for corrective osteotomy was improved using the image fusion system. This image fusion system represents an approach to reproduce the planned reduction in corrective osteotomy for distal radius fracture malunion.

## Methods

The study protocol was approved by the Institutional Review Board (No. 18–19, T2019-0178). This was a retrospective comparative study (Level of Evidence III). It has been registered as NCT03764501 at ClinicalTrials.gov (registered 21 May, 2018, retrospectively registered). This study was performed in accordance with the relevant guidelines and regulations. Informed consent was obtained from all individual participants included in the study. Ten wrists from 10 distal radius malunion patients who underwent corrective osteotomy (six females, four males, mean age 59.4 years, age range 40–78) were evaluated. The image fusion group underwent 3D preoperative planning and performed corrective osteotomy with an image fusion system (n = 5). The control group was enrolled from patients who underwent corrective osteotomy using only 3D preoperative planning (n = 5).

### Preoperative planning

**Fig. 3:**
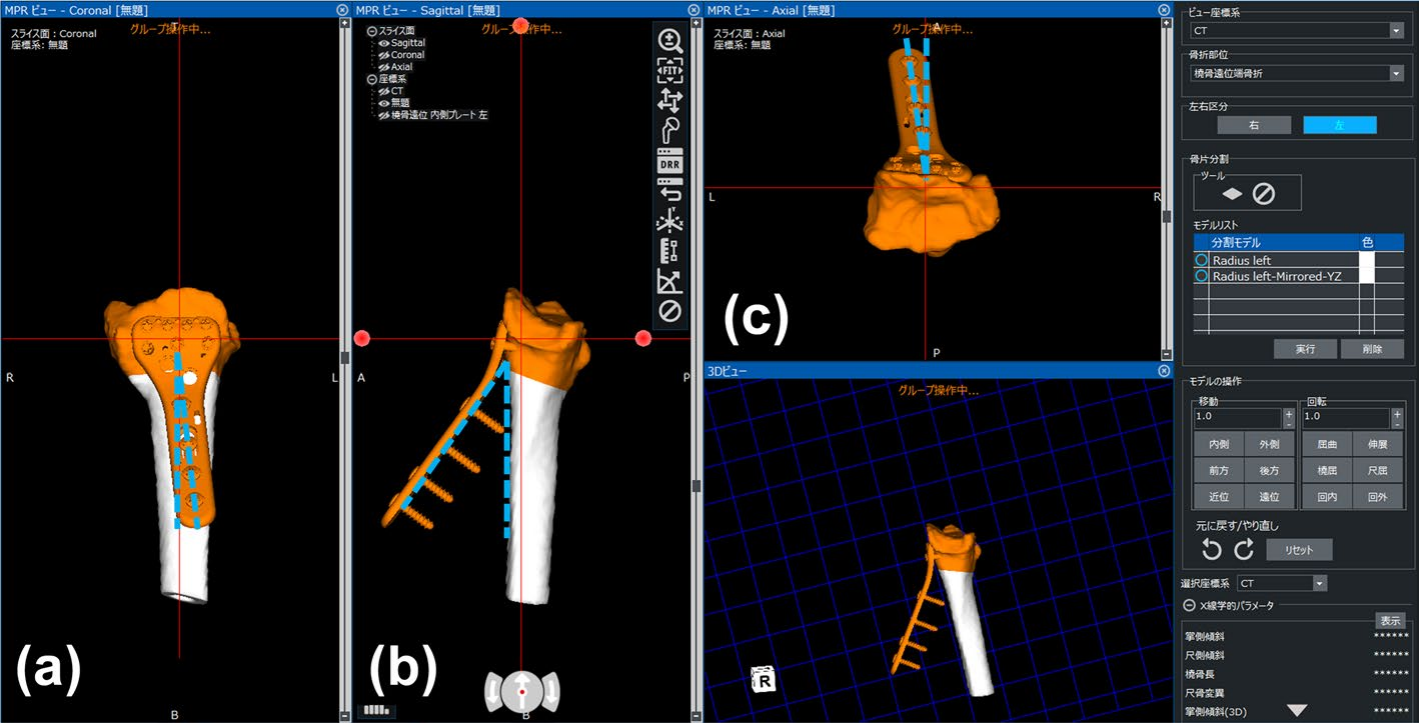
Preoperative image of plate placement. An image of plate placement was created by calculating the correction angle required to restore volar tilt, radial inclination, and rotational deformation. The plate was placed so that the distal margin was parallel to the articular surface. An image of each frame shows the plate fixed to the distal radius. The blue dotted line on the frame (**a**) shows the correction angles for the coronal view. The blue dotted line on the frame (**b**) shows the correction angles for the sagittal view. The blue dotted line on the frame (**c**) shows the correction angles for the axial view

**Fig. 4:**
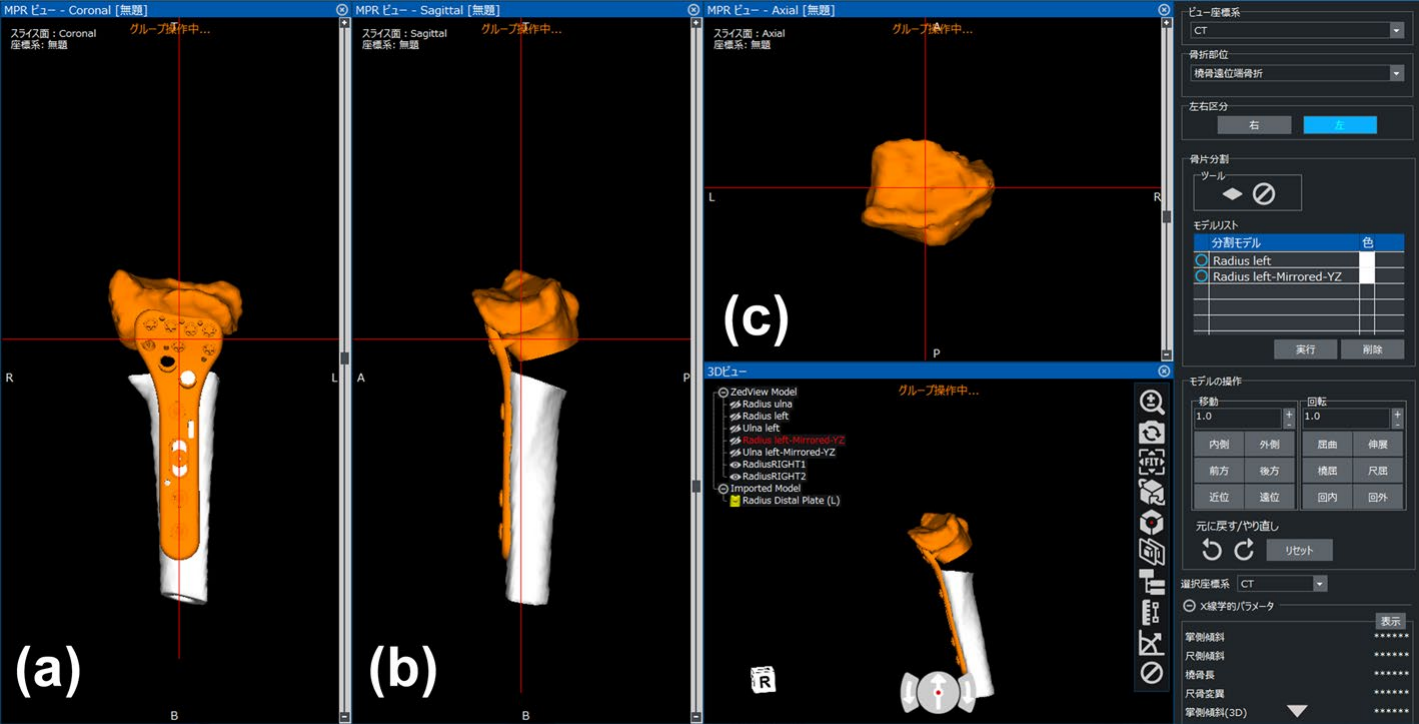
Preoperative image of reduction. After separating the distal part of the radius, reduction was achieved by fixing the proximal side of the plate to the radius shaft. The gap between the distal and proximal parts of the radius indicates the predicted bone defect. **a** Coronal view, **b** sagittal view, **c** axial view

In both groups, 3D digital preoperative planning and a surgical simulation were performed prior to surgery. Corrections to and the placement of implants were simulated using software developed by one of the authors (Zed-Trauma, LEXI Co., Ltd., Tokyo, Japan). Computed tomography (CT) of the affected and unaffected wrists using contiguous images with a slice thickness of 1–1.5 mm were taken for the simulation. Images were taken approximately 12 cm proximal to the radial joint surface. A total of 80–120 axial CT images were used for the simulation. After importing DICOM images into the software, a 3D image of the distal radius was made for both wrists. In dorsally angulated malunion, the placement of a volar locking plate was initially simulated. Computer-aided design models of different-sized implants are installed in the software; a placement image of the plate was created by calculating the correction angle required to restore volar tilt, radial inclination, and rotational deformation (Fig. 3). Stellar II locking plates (HOYA Technosurgical, Inc., Tokyo, Japan) were used in the present study. This plate system has small, medium, and large widths as well as short and long plate lengths. The plate size was selected to cover the distal radius maximally and not exceed the width of the distal radius. In addition, a sufficient length plate for the insertion of at least three screws into the radius shaft after reduction was chosen. The lengths for the distal screws were selected and a contour extraction image of the initial plate placement was saved for image fusion. The osteotomy line was set at a position that did not interfere with distal screw holes. After the osteotomy simulation, the plate and distal fragment were grouped, and the distal fragment was repositioned by adapting the proximal side of the volar locking plate to the radius shaft (Fig. 4). After repositioning the fragments, the 3D bone shape was compared with a mirror image of the unaffected radius. In the next step, simulations of the screw choices were performed for the proximal screw holes and the screw lengths for each screw hole were selected. This final reduction and implant placement image was also saved for image fusion. To compare planned and postoperative reduction shapes, preoperative plan and postoperative 3D images were created for all patients.

### *Image fusion system* and *surgical intervention*

**Fig. 5:**
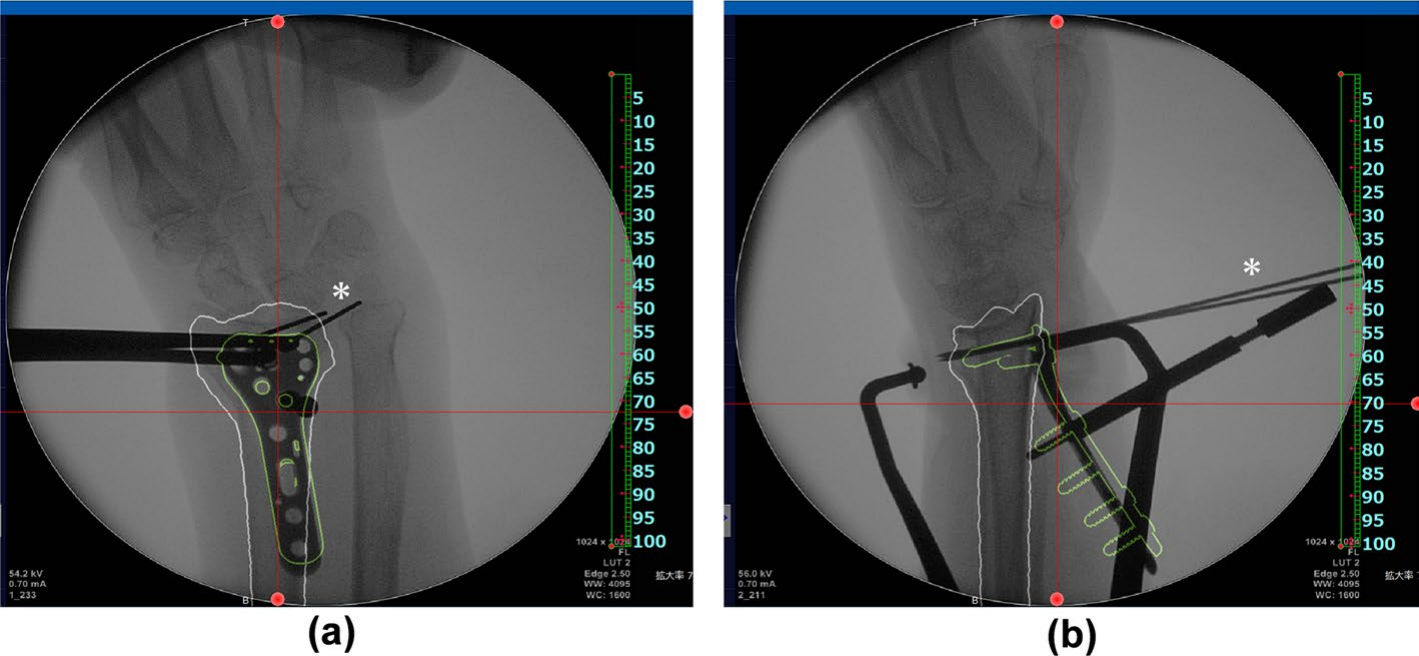
Fusion image of the preoperative plan and fluoroscopy for plate placement. Based on the plate placement image, a contour extraction image was created and displayed on the fluoroscopy image for surgery. The plate was placed to fit the contour line. **a** Anterior–posterior view, **b** lateral view. * shows temporary fixing wires

**Fig. 6:**
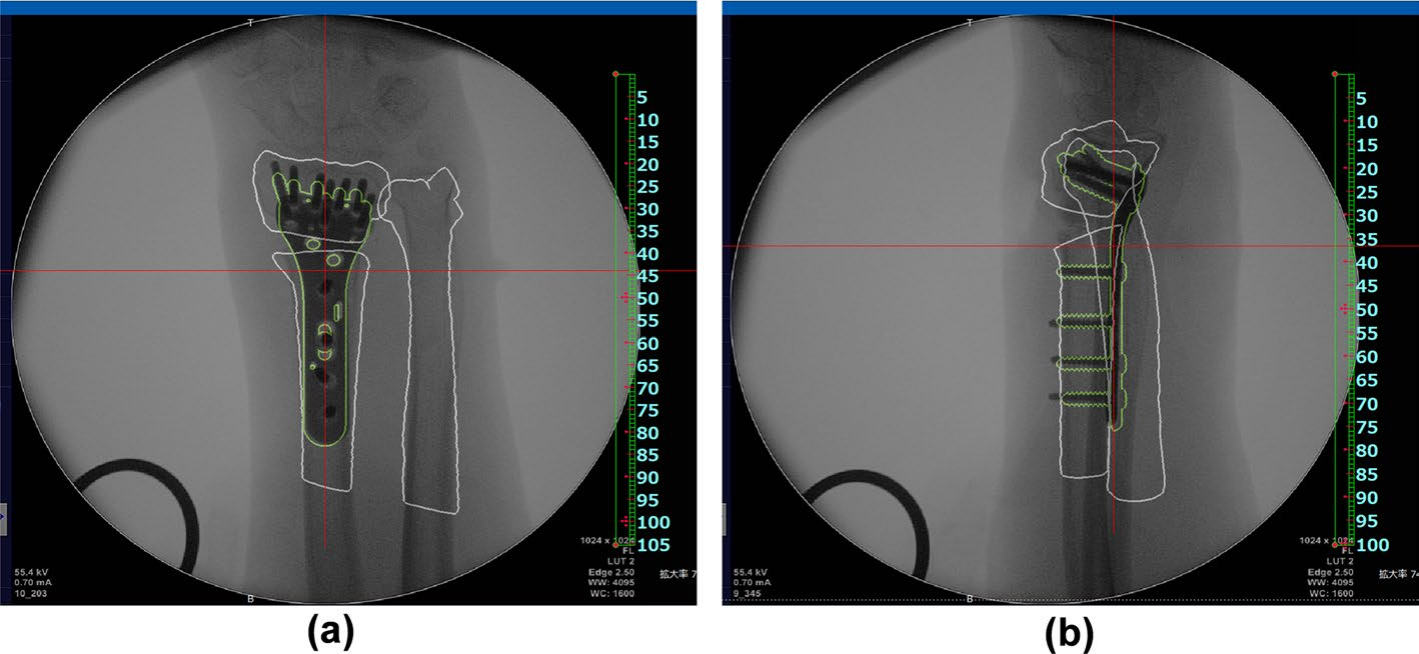
Fusion image of the preoperative plan and fluoroscopy for reduction. Based on the reduction image, the distal fragment was repositioned by adapting the proximal side of the volar locking plate to the contour extraction image

Regarding image fusion, 3D images of preoperative plans were converted to digitally reconstructed radiographs. Bone and implant contour extraction images were created for anterior–posterior and lateral views based on 3D images. Fusion images were displayed on a monitor overlapping the outline of the 3D preoperative plan and the fluoroscopic image. Corrective osteotomy was performed under general anesthesia. In the image fusion group, the outline of the planned image was displayed on a monitor overlapping the fluoroscopy image during surgery. Before starting surgery, the contour extraction image size was calibrated by measuring a known length. A surgeon performed corrective osteotomy based on the fusion image. Before osteotomy, a plate placement image was displayed on the monitor (Fig. 5). In the first step, the plate was placed on the distal radius according to the outline of the plate image and fixed with two temporary fixing wires. Outlines of the anterior–posterior and lateral views were used. According to the direction of the fluoroscopic image, the direction of the contour image was changed to the anterior–posterior or lateral view. Plate placement was checked with a fusion image for each direction. After determining the plate position at the distal radius, distal screw holes were pre-drilled. The plate was then removed leaving the temporary fixing wires. In the second step, osteotomy was performed at a level that did not interfere with the distal screws of the plate. In the third step, the plate was returned to the originally selected position under the guide of the temporary fixing wires, and the distal screws were inserted into the pre-drilled holes. Finally, the distal fragment was repositioned by adapting the proximal side of the volar locking plate to the radius shaft, and the plate was fixed with screws (Fig. 6).

In the control group, the surgeon performed reduction and placement of the plate while comparing separate images of the 3D preoperative plan and fluoroscopy during surgery. In both groups, a beta-tricalcium phosphate-based artificial bone or autologous bone graft was performed depending on the size of the bone defect. Surgeries were performed by one hand surgeon.

### Evaluations

**Fig. 7:**
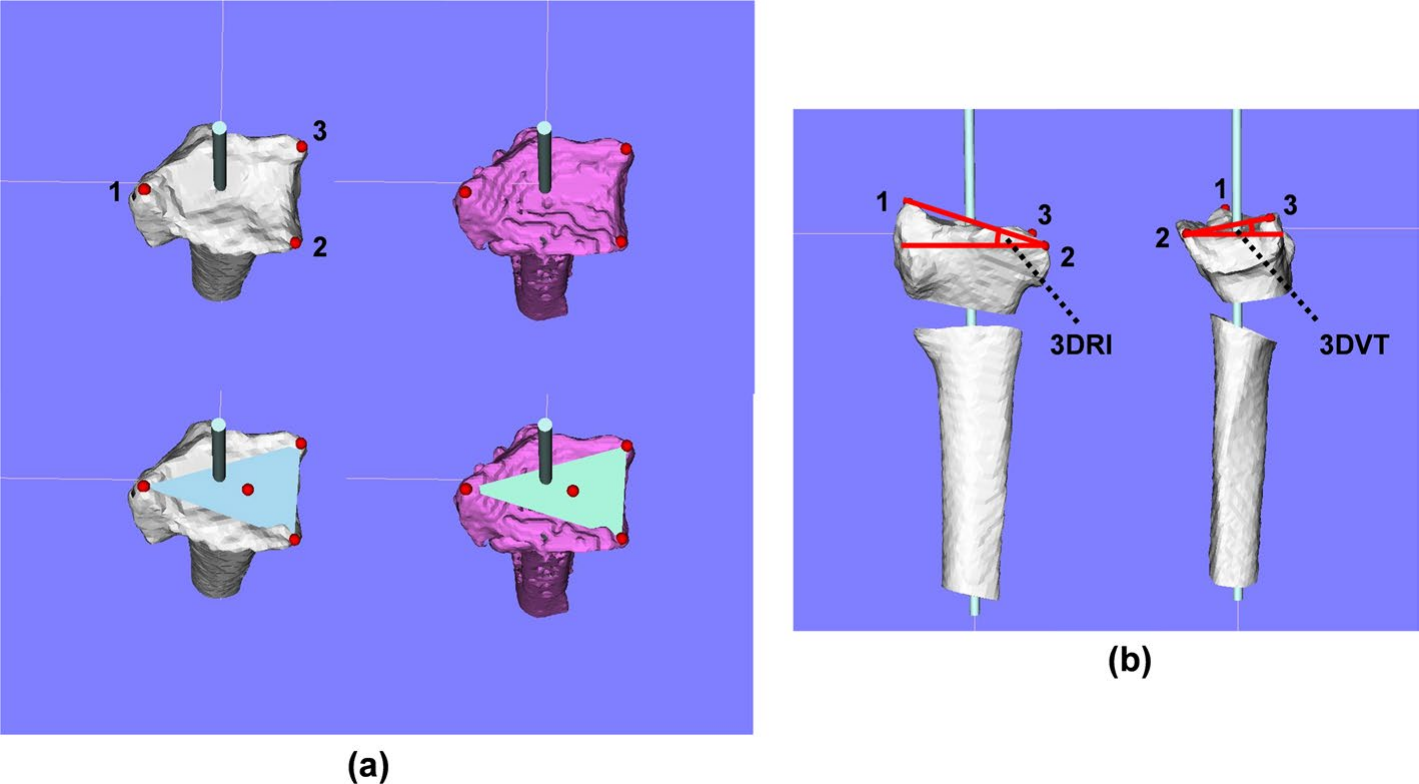
Example images of 3D reference points. **a** An example image for each reference point. Left row images show the reference points in the preoperative plan image. Right row images show the reference points in the postoperative image. The light blue bar indicates the long axis of the distal radius. Red dots indicate the radial styloid process: reference point (1), the sigmoid notch volar edge: reference point (2), the sigmoid notch dorsal edge: reference point (3), and the barycentric coordinates. **b** An example image for 3DVT and 3DRI. The angle between a connecting line from reference point (2) to reference point (3) and a line perpendicular to the longitudinal axis of the radius was measured as the volar tilt on a 3D image in the sagittal view (3DVT). The angle between a line from reference point (1) to reference point (2) and a line perpendicular to the longitudinal axis of the radius was measured as the radial inclination on a 3D image in the coronal view (3DRI). The measured angles in the example image were 19.2° and 12.7° for 3DRI and 3DVT, respectively

Pre- and post-operative 3D images of the distal radius were analyzed using image analysis software (BoneSimulater, Orthree, Osaka, Japan). DICOM data of CT images were imported into the software. A 3D surface model of the radius was constructed with a surface construction algorithm. The long axis of the radius was calculated from the 3D surface model of the intact part of the preoperative distal radius image. Image registration for the preoperative plan and postoperative reduction were performed using the intact part of the distal radius image. The y-axis was defined as the long axis of the radius, and the proximal direction was defined as positive. The z-axis was parallel to the orthogonal projection of a line initiating at the base of the distal ulnar sigmoid notch and continuing to the radial styloid process on a plane perpendicular to the y-axis. The radial direction on the z-axis was defined as positive. The x-axis was normal to the yz plane and the palmar direction was defined as positive. The yz, xy, and xz planes were defined as the coronal, sagittal, and axial planes, respectively. The origin of coordinates was defined as the intersection of the joint surface and the radius of the long axis on the preoperative plan image. Three reference points: (1) the radial styloid process, (2) the sigmoid notch volar edge, and (3) the sigmoid notch dorsal edge, were marked on pre- and post-operative 3D images (Fig. 7). The 3D coordinates of each reference point and the barycentric coordinates of the plane connecting the three reference points were evaluated using the 3D images of the preoperative plan and postoperative reduction.

In the sagittal view, the angle between a connecting line from reference point (2) to reference point (3) and a line perpendicular to the longitudinal axis of the radius was measured as volar tilt (3DVT). In the coronal view, the angle between a line from reference point (1) to reference point (2) and a line perpendicular to the longitudinal axis of the radius was measured as radial inclination (3DRI).

In evaluations of clinical outcomes, Mayo wrist scores [27] were recorded 3 and 6 months after surgery.

### Statistical analysis

Results are expressed as the mean ± standard deviation. Distances between the preoperative plan and postoperative reduction for each reference point were measured for both groups. Differences between the preoperative plan and postoperative reduction for 3DRI and 3DVT were measured for both groups. The Shapiro–Wilk test was used to test the normality of datasets. The distances of reference points and differences in angles were compared using Welch’s *t*-test. The Mann–Whitney U test was used for unevenly distributed datasets (3DVT). P values of less than 0.05 were considered to be significant. All analyses were performed using BellCurve for Excel version 2.12 (SSRI Co., Tokyo, Japan).

## Data Availability

The datasets used and/or analyzed during the present study are available from the corresponding author upon reasonable request.

## Abbreviations

3D: Three-dimensional
2D: Two-dimensional
DICOM: Digital imaging and communications in medicine
CT: Computed tomography
3DVT: Three-dimensional volar tilt
3DRI: Three-dimensional radial inclination
